# Evaluation of four DNA extraction kits for implementation of nanopore sequencing in routine surveillance of antimicrobial resistance in low-resource settings

**DOI:** 10.3389/fmicb.2025.1715467

**Published:** 2025-11-25

**Authors:** Natasia R. Thornval, Niamh Lacy-Roberts, Pernille Nilsson, Carmen Espinosa-Gongora, Henrik Hasman, Joana Mourão, Astrid Rasmussen, Ana Rita Rebelo, Christa Gibson, Rene S. Hendriksen

**Affiliations:** 1 National Food Institute, Technical University of Denmark, Kongens Lyngby, Denmark; 2 National Reference Laboratory for Antimicrobial Resistance, Department of Bacteria, Parasites and Fungi, Statens Serum Institut, Copenhagen, Denmark

**Keywords:** antimicrobial resistance, WGS, nanopore sequencing, capacity building, surveillance, pathogens, GLASS, low-resource settings

## Abstract

**Introduction:**

Whole genome sequencing (WGS) is a valuable tool in surveillance of antimicrobial resistance (AMR). However, the technology faces several implementation challenges in low-resource settings. While advances in Oxford Nanopore Technologies (ONT) field sequencing have enabled sequencing in low-resource settings, DNA extraction remains a critical barrier to implementation.

**Methods:**

We evaluated four commercially available DNA extraction kits: QIAGEN DNeasy Blood & Tissue, NEB Monarch® HMW, Thermo Fisher MagMAX™ Microbiome, and Thermo Fisher MagMax™ Viral/Pathogen, for their suitability in ONT-based AMR surveillance across Gram-positive and Gram-negative bacterial strains. Kits were evaluated for DNA purity, yield, and fragment length, as well as sequencing metrics including mean read quality, read N50, sequencing depth, multilocus sequence typing concordance, and AMR gene detection. Practical parameters such as cost, hand-on time, and equipment requirements were also assessed.

**Results:**

The DNeasy Blood & Tissue kit consistently produced DNA of sufficient quality and quantity to enable high-sequencing depth ONT sequencing, enabling robust multi-locus sequence typing and AMR gene recovery, while remaining cost-effective and requiring minimal technical expertise.

**Discussion:**

These findings support the integration of optimized DNA extraction workflows into public health surveillance systems. The DNeasy Blood & Tissue kit offers a reliable and scalable solution for real-time genomic monitoring of antimicrobial resistance in resource-limited settings.

## 1 Introduction

Antimicrobial resistance (AMR) poses a critical global health challenge, particularly in low-and-middle income countries (LMICs) across Africa, where the highest mortality rates from AMR-related causes are observed (Murray et al., 2022). LMICs are disproportionately affected by AMR due to a higher burden of communicable diseases, excessive use of antibiotics, and poor to insufficient access to antibiotics in the same geographical locations (Aruhomukama, 2022; Laxminarayan, 2022).

In many high-income countries, AMR surveillance using whole-genome sequencing (WGS) technologies is well-established, providing real-time, actionable data that informs public health interventions and clinical management (Karp et al., 2017; Sia et al., 2021; Jarocki et al., 2024). By contrast, WGS for surveillance of AMR and endemic diseases remains underutilized in Africa due to key challenges including difficulties procuring sequencing equipment and consumables, lack of robust supply chains, and limited stable storage conditions for reagents, especially in remote areas (Kekre et al., 2021; Konono et al., 2024; Mukhwana et al., 2024). Despite these challenges, WGS has demonstrated clear value during outbreaks in African countries as seen during the 2014–2016 West-African Ebola epidemic (Quick et al., 2016). However, WGS is especially important in AMR surveillance, because resolving both the pathogen and the resistance mechanisms involved has direct applications for treatment, stewardship, and infection control (Karp et al., 2017; Hendriksen et al., 2019; Wee et al., 2020; Baker et al., 2023; Sherry et al., 2025).

The MinION sequencing device from Oxford Nanopore Technologies (ONT) offers a laptop-compatible setup with relatively low startup cost, portability, and minimal spatial requirements. These features make MinION sequencing highly adaptable to environments with limited infrastructure, laboratory space and making it suitable for diagnostic and surveillance applications in regions with intermittent power. Consequently, ONT offers a potential solution to many of the challenges in expanding WGS capacity in Africa, enabling frontline diagnostics and surveillance at sentinel sites (Aruhomukama, 2022; Lu et al., 2022). Until recently, the error rates associated with ONT sequencing were too high for routine AMR surveillance, particularly in instances requiring high accuracy to detect specific gene variants conferring to different phenotypes. Recent advances in ONT’s chemistry have reduced error rates, and emerging studies suggest ONT may even outperform the widely used Illumina short-read technology in the identification of AMR genes (Sereika et al., 2022; Lohde et al., 2024; Sierra et al., 2024; Prior et al., 2025). While ONT sequencing itself is highly scalable in LMIC settings, the DNA extraction and library preparation often require highly specialized laboratory technicians and expensive auxiliary equipment. However, obtaining high-quality DNA in adequate quantity is crucial for successful sequencing and analysis. Several studies have evaluated DNA extraction workflows for ONT sequencing, however, to our knowledge, none have specifically addressed the challenges of implementing a robust method in low-resource settings for routine surveillance of a broad range of different species (De Maio et al., 2019; Gand et al., 2023; Kruasuwan et al., 2024). Overcoming these challenges is essential if ONT-based WGS is to be successfully implemented for routine AMR surveillance (Eagle et al., 2023; Konono et al., 2024).

The UK Aid program Fleming Fund Regional Grant “SeqAfrica” was established in 2019 with the main objective of developing, expanding and supporting WGS and bioinformatics capacity for AMR surveillance across Africa. In preparation for rolling out ONT WGS to SeqAfrica sentinel sites, we systematically compared four commercial DNA extraction kits using both Gram-positive and Gram-negative isolates, covering different lysis strategies (enzymatic, chemical, and bead-beating) and purification methods (silica spin-column, magnetic bead-based, and glass bead-based). Our primary outcome was AMR gene detection performance in an ONT workflow, with secondary outcomes including MLST concordance, sequencing depth, and practical feasibility (cost, hands-on time, and equipment requirements).

## 2 Materials and methods

### 2.1 Bacterial strains

One Gram-positive and two Gram-negative bacterial species relevant to the World Health Organization (WHO) Global Antimicrobial Resistance and Use Surveillance System (GLASS) (World Health Organization, 2020) and Food and Agriculture Organization of the United Nations (FAO) Priority pathogens (FAO, 2021), and a Coagulase-negative Staphylococci, were selected serving as reference strains for the evaluation of four commercially available DNA extraction kits: *Escherichia coli* GENOMIC22-004, *Campylobacter coli* GENOMIC20-006, *Staphylococcus hominis* NT-2025, and *Enterococcus faecalis* GENOMIC22-006 (Table 1). The *S. hominis* NT-2025 strain was included in this study because it has been described as a particularly difficult strain to lyse (de Oliveira et al., 2014; Pushkaran et al., 2015).

**Table 1:** Overview of strains in this study.

Strain name	Species	NCBI accession number	Sequence type (ST)	Total base pairs (bp)	No. of plasmids	AMR genes
GENOMIC22-004	*Escherichia coli*	GCA_029094485	410	5,164,118	6	*rmtC*, *aac(6′)-lb3*, *bla*_CMY-6_, *bla*_NDM-1_, *bla*_OXA-181_, *qnrS1*, *sul1*
GENOMIC20-006	*Campylobacter coli*	GCA_949361535	3,336	1,814,825	1	*aadE-Cc, bla*_OXA-193_, tet(O)
GENOMIC22-006	*Enterococcus faecalis*	GCA_029167565	6	3,407,461	3	*aac(6′)-aph(2″), lsa(A), erm(B), cat(pC221), tet(M), VanHBX*
NT-2025	*Staphylococcus hominis*	JBRFSD000000000	223	2,337,290	4	*aadD, blaZ, fusB, tet(k), bleO*

Strains were cultured from frozen stock on TSA agar plates w/5% calf blood (SSI Diagnostica, Hillerød, Denmark). *C. coli* strain was incubated overnight at 41 ± 1 °C under microaerobic conditions in a Tri-Gas incubator (10% CO2, 5% O2, 85% N2), and all the other isolates at 37 ± 1 °C in ambient air.

High-quality complete closed reference genomes of the four strains were available for the comparative analysis of this study with genome sizes varying from 1.81 Mbp to 5.16 Mbp (Table 1). The reference genomes were generated through PacBio sequencing or from hybrid-assemblies based on Illumina and ONT sequences and available at NCBI (Table 1). These high-quality closed genomes served as references for assessing the results obtained from testing the DNA extraction kits on the same isolates.

### 2.2 DNA extraction methods

**Figure 1:**
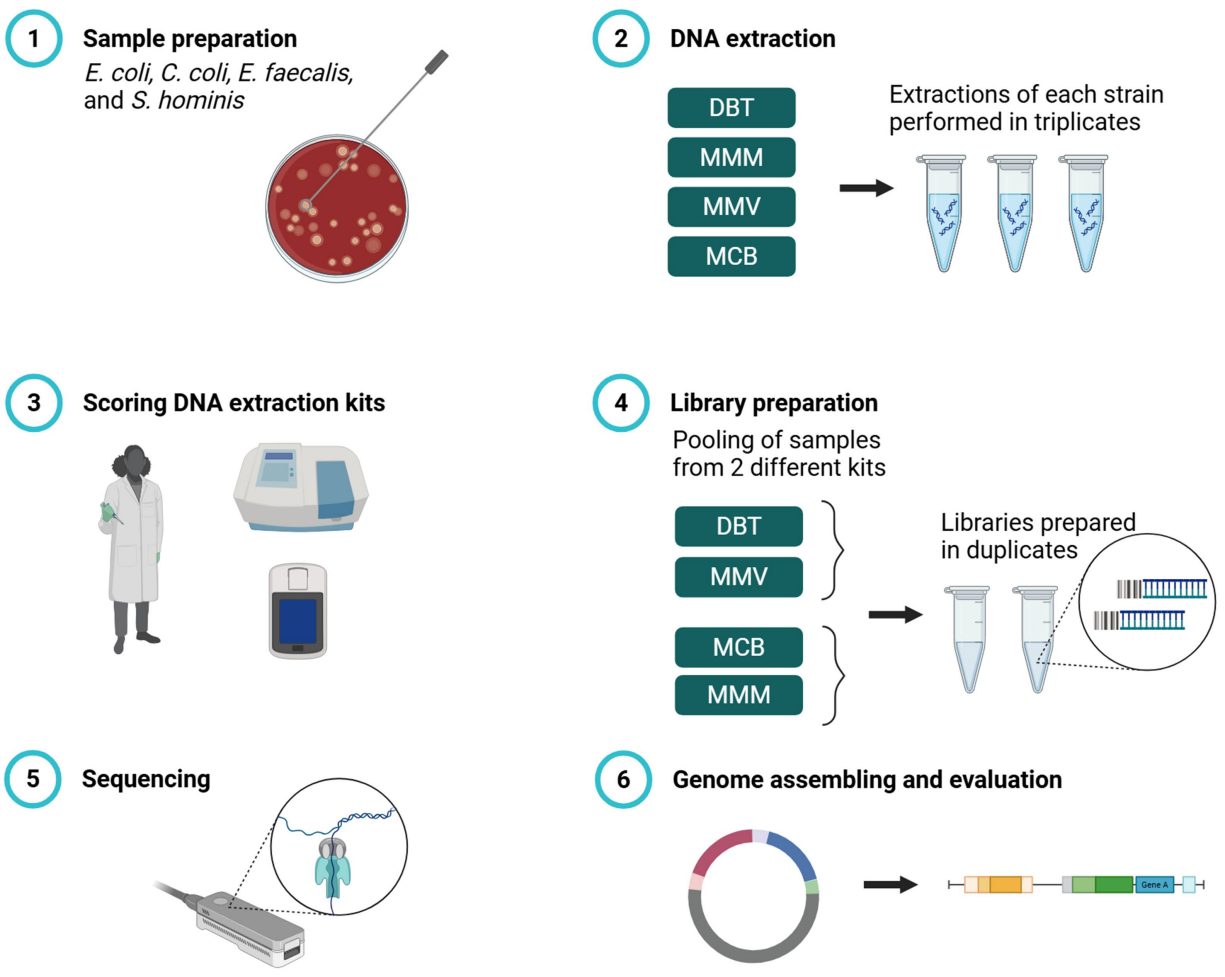
Workflow for evaluation of four commercial DNA extraction kits for Nanopore WGS on Gram-positive and Gram-negative bacteria. (1) Pure cultures of four bacterial strains: *Escherichia coli*, *Campylobacter coli*, *Enterococcus faecalis*, and *Staphylococcus hominis*. (2) gDNA from all four strains was extracted in triplicates to a total of 12 reactions with DNeasy Blood & Tissue spin-columns (DBT), MagMAX™ Microbiome Ultra Nucleic Acid Isolation kit (MMM), MagMAX™ Viral/Pathogen Ultra Nucleic Acid Isolation kit (MMV), and Monarch® HMW DNA Extraction kit for Tissue (MCB) according to manufacturer’s protocol. (3) DNA extraction kits were scored according to ease of use, time per sample, cost, DNA concentration, and DNA purity. (4) During library preparation, triplicate extractions per strain were pooled, combining DBT with MMV and MCB with MMM. Libraries were prepared in duplicate using Rapid Barcoding Kit V14 and sequenced on R10.4.1 Flow Cells (Oxford Nanopore Technologies). Created in https://BioRender.com.

Genomic DNA (gDNA) was extracted in triplicate for each bacterial strain using four different commercially available DNA extraction kits: DNeasy Blood &Tissue spin-columns (DBT) (Qiagen, Germany), MagMAX™ Microbiome Ultra Nucleic Acid Isolation kit (MMM) (Thermo Fisher Scientific, USA), MagMAX™ Viral/Pathogen Ultra Nucleic Acid Isolation kit (MMV) (Thermo Fisher Scientific, USA), and Monarch® HMW DNA Extraction kit for Cells & Blood (MCB) (New England Biolabs, USA). The kits spanned distinct lysis strategies (enzymatic, chemical, and bead-beating) and purification methods (silica spin-column, magnetic beads, and glass beads) (Table 2). DNA from a third of a 10-μl loop of bacterial mass was extracted for each of the four reference strains in triplicate according to the manufacturers’ protocol (Figure 1). The manufacturers’ protocols were amended for all post-lysis steps which required vortexing. The vortexing steps were replaced with gently flicking the tube and pipetting up-and-down to mix. For DBT, MCB, and MMM lysostaphin (15 mg/ml) was added during lysis of the *S. hominis* strain. The laboratory scientist performing the DNA extractions practiced two times with each method before the study.

**Table 2:** Summary of four different commercially available DNA extraction kits.

Abbreviations	**DNA extraction kit**	**Manufacturer**	**Lysis method** ^**1**^		**Purification method**	**Additional reagents required** ^**2**^		**Laminar flow bench required**	**Non-standard equipment required** ^**3**^
			**Gram-negatives**	**Additional lysis for Gram-positives**		**Gram-negatives**	**Gram-positives**		
DBT	DNeasy Blood & Tissue spin-columns	Qiagen, Germany	Chemical	Chemical + enzymatic digestion	Silica spin-column	No	Yes	No	No
MCB	Monarch® HMW DNA Extraction Kit for Tissue	New England Biolabs, USA	Chemical	Chemical + enzymatic digestion	Glass beads	Yes	Yes	Yes	Microcentrifuge with cooling (4°C)
MMM	MagMax™ Microbiome UltraNucleic Acid isolation kit	Applied biosystems, USA	Bead beating + chemical		Magnetic beads	No	No	No	No
MMV	MagMAX™ Viral/Pathogen Ultra Nucleic Acid Isolation Kit	Applied biosystems, USA	Enzymatic digestion		Magnetic beads	No	No	No	No

Abbreviations for the names of the kits, name of the kit and manufacturer, and DNA extraction method used with the kit (silica spin column, magnetic beads, or glass beads).

### 2.3 DNA quantity and quality assessment

Extracted gDNA concentrations were measured by Qubit 4 Fluorometer (Invitrogen, Thermo Fisher Scientific, Waltham, MA, USA) using the Qubit High Sensitivity Quantification Kit (Invitrogen, Thermo Fisher Scientific), except for concentrations >120 ng/μl where the Invitrogen Qubit Broad Range Quantification kit (Invitrogen, Thermo Fisher Scientific) was used according to the manufacturer’s protocol. Yield was reported as ng dsDNA/μl in final elution across the four different DNA extraction kits. Sample purity was measured with the NanoPhotometer NP80 Mobile (Implen GmbH, München, Germany) and reported in the A_260/230_ and A_260/280_ ratios.

### 2.4 Sequencing, *de novo* assembly, and data analysis

To evaluate the effect of the four DNA extraction methods on WGS performance, the triplicate extractions of each method (*n* = 12) were used for library preparation using ONT’s Rapid Barcoding Kit with the v14 chemistry (SQK-RBK114, Oxford Nanopore Technologies, Oxford, UK) according to the manufacturer’s protocol (RBK_9126_v110_revO_24Mar2021), using 50 ng input gDNA per sample. During library preparations, samples from two methods were pooled (DBT with MMV; MCB with MMM), yielding pooled sets of 24 barcoded samples. Each library was prepared in duplicate, producing four multiplexed libraries in total. Libraries were loaded on R10.4.1 flow cells (Oxford Nanopore Technologies) with more than 1,200 active pores pre-run (Figure 1). Flow cells were sequenced on the GridION platform (Oxford Nanopore Technologies) and MinKNOW v23.11.4 was used to perform real-time basecalling with default settings except that minimum read length was set to 400 bp using the super-accurate (SUP) basecalling model.

Fastq files were concatenated post demultiplexing per barcode with no further quality filtering. Nanostat v1.6.0 (De Coster et al., 2018) was used to generate quality metrics (i.e., read N50, mean read quality, number of reads, and total bases). Genomes were assembled with Flye v2.9.1 (Kolmogorov et al., 2020) using default settings. NCBI accession numbers for raw sequencing data can be found in supplementary material Table S3.

Multilocus sequence typing (MLST) for each of the four reference genomes and the assemblies from this study were determined using an *in-house* pipeline and MLST v2.0 (Larsen et al., 2012) with 100% identity match and 100% coverage. A new MLST type for *S. hominis* strain was generated through submission of the reference genome to PubMLST (Jolley et al., 2018). AMR genes were detected using ResFinder v4.2.3 database version 2.3.2 (Camacho et al., 2009; Bortolaia et al., 2020) with ≥95% identity and ≥95% coverage.

### 2.5 Time and cost estimation

The prices of the four commercially available DNA extraction kits were calculated on a per-reaction basis, using pricing information from local Danish distributors as of January 2025, assuming bulk purchases of between 50 and 100 reactions. The total cost per sample included costs of reagents and consumables not included in the kits, (i.e., pipette tips, tubes, chemicals, enzymes), calculated in Euro (EUR) per sample without value added tax (VAT). The cost associated with addition of lysostaphin, and any auxiliary equipment was not included in any calculations. Hands-on time and total processing time were recorded based on the extractions of 12 isolates for both Gram-positive and Gram-negative bacteria. Hands-on time excluded incubation steps lasting ≥10 min, which were instead included in the total time. Single-time preparation steps, such as ethanol addition to reagents or lysis buffer preparation for Gram-positive bacteria, were not included in hands-on time calculations.

### 2.6 Performance assessment

All statistical analysis and visualizations were performed in R Statistical Software v4.4.2 (R Core Team, 2021). To assess the success of the DNA extraction methods, the yield was measured as ng/μl in final elution and purity measured as A_260/230_ and A_260/280_. Purity of samples with DNA concentrations below 10 ng/μl was not included due to decreased accuracy of measurements (Thermo Fisher Scientific, 2020). Measurements were reported in mean ± standard deviation (SD) across replication for triplicate extraction according to strain and DNA extraction method.

Mean and SD for yield, absorbance, mean read quality (Q-score), read N50, and sequencing depth (mean base coverage) were calculated and visualized for each strain and DNA extraction method. Assemblies with a total genome length differing more than 10% from the closed reference genome were defined as failed. Significant effects on quality measurements for the four different strains were assessed with the non-parametric Kruskal Wallis test for each DNA purification method, as data did not meet assumption of normality. Significant results were further analyzed with *post hoc* Dunn tests with Benjamini-Hochberg correction using the FSA package v0.9.6 (Ogle et al., 2025). For both tests, a statistical significance threshold of *p* < 0.05 was applied.

A binary outcome variable was created to define successful WGS. Assemblies with ≥30× mean depth, and detection of all seven MLST housekeeping genes and all AMR genes according to the reference genomes were assigned a value of 1 (Supplementary Tables S1, S3–S5). Assemblies were assigned a 0 if any of these outcomes failed. As some extraction methods had zero successes, the effect of the DNA extraction method on WGS success was investigated by Firth’s penalized logistic regression analysis using the logistf v.1.26.1 package (Heinze et al., 2025). The final model only included DNA extraction method as a covariate as the strains did not improve model fit when tested by likelihood ratio test. Significance was defined as *p* < 0.05.

## 3 Results

### 3.1 Pre-sequencing performance

**Figure 2:**
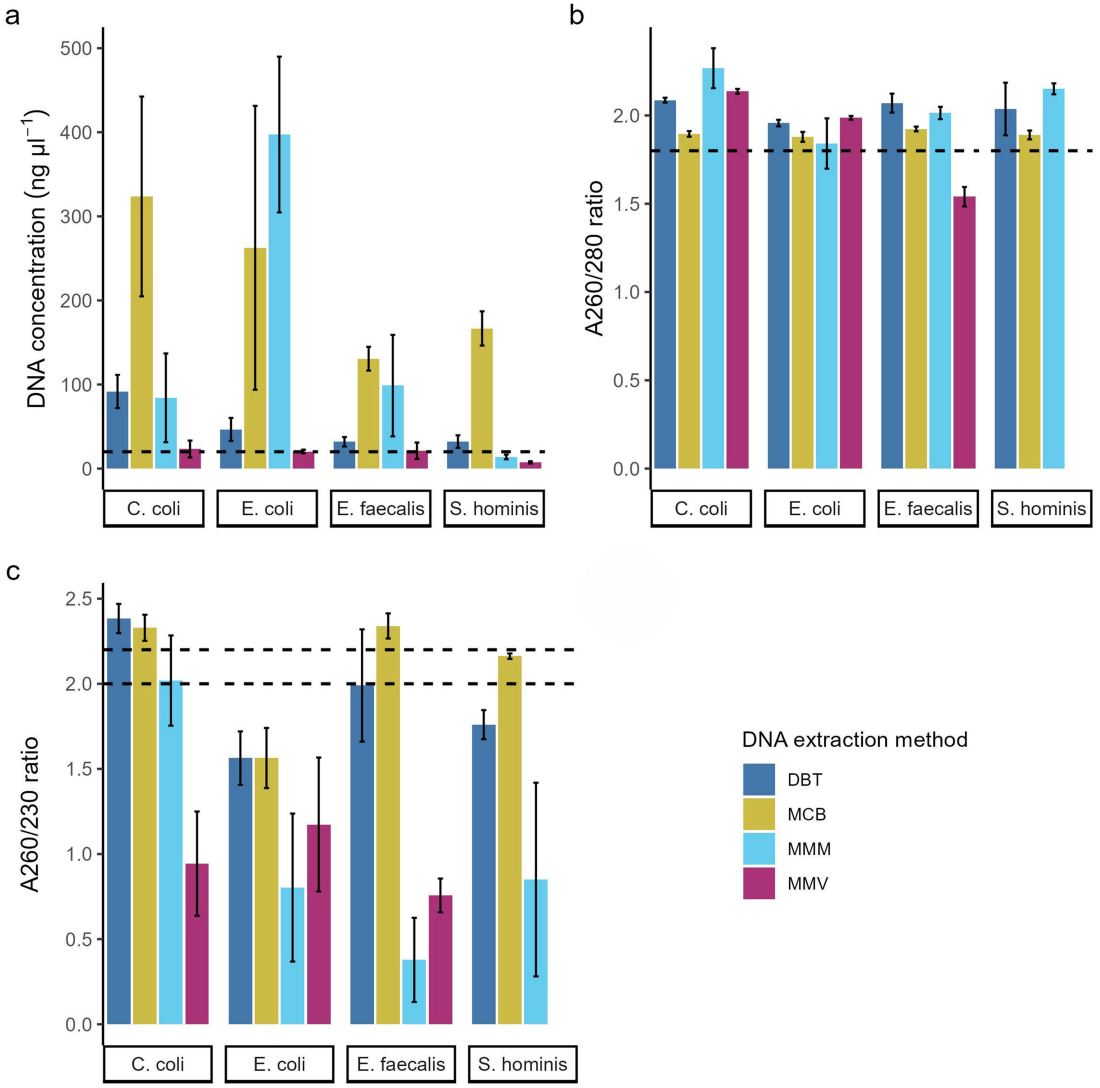
Effect of DNA extraction methods. **(a)** DNA concentration (ng/μl), DNA purity measured by **(b)** A_260/280_ ratio and **(c)** A_260/230_ ratio of the four bacterial species. The bar charts show mean across replications ± SD of three replications. Dotted line indicates **(a)** the DNA concentration required to provide 200 ng gDNA for the current Nanopore library preparation protocol for the Rapid Barcoding kit (RBK_9176_v114_revR_04Jun2025) and **(b,c)** the recommended purity measurements from Nanopore. DNeasy Blood & Tissue spin-columns (DBT, dark blue), Monarch® HMW DNA Extraction kit for Tissue (MCB, yellow) MagMAX™ Microbiome Ultra Nucleic Acid Isolation kit (MMM, light blue), and MagMAX™ Viral/Pathogen Ultra Nucleic Acid Isolation kit (MMV, purple).

The four bacterial strains (Table 1) were processed for DNA extraction in triplicate using four different commercially available DNA extraction kits (Table 2). The concentration of extracted gDNA was measured to evaluate if the extractions yielded at least 5 ng/μl, which is required for 50 ng gDNA in 10 μL input for the Rapid-Barcoding kit protocol. Across all four strains, each kit produced sufficient DNA concentrations in all replicates. However, according to most recent recommendations from ONT (RBK_9176_v114_revR_04Jun2025), 20 ng/μl in a 10 μL input (200 ng input DNA) is now required. The DBT and MCB kits met these updated requirements, whereas lower concentrations were obtained with the MMV in all the *S. hominis* strain replicates (5.66 ng/μl – 8.48 ng/μl) and by MMM in replicates from all four species (Figure 2a).

In terms of purity of the extracted DNA, A_260/280_ ratios ranged from 1.44–2.40 with significant differences between the DNA extraction methods across the four species (*p* < 0.02). MMV exhibited a lower A_260/280_ ratio for the *E. faecalis* strain (1.54 ± 0.05, *p* < 0.006) and did not produce sufficient concentrations of DNA for the *S. hominis* strain to accurately measure purity(Koetsier and Cantor, 2019). MMM produced a higher ratio for *C. coli* GENOMIC20-006 (2.27 ± 0.11) compared with the recommended ratio. Methods MCB and DBT yielded > 1.8 A_260/280_ for all four strains, although multiple DBT measurements were above 2.0. All replicates were within the recommended range when using the MCB method (A_260/280_ = 1.84–1.93) (Figure 2b and Table 3).

**Table 3:** Comparison of performance of four commercially available DNA extraction kits.

Parameters		DNeasy Blood & Tissue (DBT)	Monarch® HMW DNA Extraction Kit for Tissue (MCB)	MagMax™ Microbiome UltraNucleic Acid isolation kit (MMM)	MagMAX™ Viral/Pathogen Ultra Nucleic Acid Isolation Kit (MMV)
Cost per sample (EUR)^1^ (Additional cost associated with Gram-positives)		6.10 (0.10)	13.50 (0.21)	8.22 (0)	8.08 (0)
Hands-on time (minutes)^2^		83	134	169	86
Total time incl. Incubation in (minutes)^2^ (additional time associated with Gram-positives)		143 (30)	224 (50)	219 (0)	196 (0)
Non-Standard equipment		No	Requires refrigerated centrifuge and laminar airflow bench	No	No
DNA yield (ng/μl)		High (> 20 ng/μl for all replicates)	High (> 20 ng/μl for all replicates)	Variable (< 20 ng/μl some replicates across all four strains)	Low (<20 ng/μl)
A_260/280_^3^	Mean ± SD	2.04 ± 0.09	1.90 ± 0.03	2.07 ± 0.18	1.89 ± 0.26
	Range	1.85–2.17	1.84–1.93	1.66–2.41	1.44–2.15
A_260/230_^3^	Mean ± SD	1.92 ± 0.36	2.10 ± 0.34	1.01 ± 0.73	0.96 ± 0.33
	Range	1.38–2.47	1.34–2.43	0.12–2.19	0.63–1.55
Read quality (*Q*-score)		15.83 ± 0.80	15.26 ± 0.57	14.30 ± 0.83	13.23 ± 1.23
Read N50 (bp)		8,151 ± 4,151	12,105 ± 2,996	8,681 ± 5,238	3,168 ± 1,480
Sequencing depth (×)		99 ± 68	82 ± 62	57 ± 44	6 ± 8
WGS success rate		100% (24/24)	87.50% (21/24)	70.83% (17/24)	0% (0/24)

^1^ The prices of the kits are calculated per sample if bought in packages of 46 and 100 reactions in Denmark and included costs of reagents not included in kit (i.e., pipette tips, tubes, chemical, enzymes), calculated in EUR per sample. Any potential lysostaphin used was excluded from total cost. All prices were pre-tax totals as of Jan 2025. The startup cost for auxiliary equipment and any other running cost associated was not included in cost per sample. ^2^ Hands-on and total time are measured as time spent for 12 reactions. ^3^ Measurements from samples with DNA concentration below 10 ng/μl due to inaccuracy of measurements.

For the A_260/230_ ratios, no method had all replicates within the ONT recommended range of 2.0–2.2, however the DNA extraction methods had a significant effect on the ratio across all strains (*p* < 0.002). The magnetic bead-based methods MMM and MMV exhibited lower A_260/230_ ratios with reduced reproducibility. MMV exhibited low A_260/230_ ranges across all four strains (0.96 ± 0.33). MMM performed poorly overall (1.01 ± 0.73) but yielded values close to the recommended range (2.02 ± 0.26) for all replicates of the *C. coli* strain. The MCB and DBT methods produced lower values for the *E. coli* strain (Figure 2c) compared with the other species. While DBT had significantly higher A_260/230_ values than the magnetic bead-based methods (MMM and MMV) for the Gram-negatives and *S. hominis* strain (*p* < 0.04), the Gram-positive replicates were slightly lower and less reproducible (*E. faecalis* GENOMIC22-006: 1.99 ± 0.33; *S. hominis* NT-2025: 1.76 ± 0.09). MCB gave acceptable ratios for all Gram-positive and *C. coli* replicates (2.14–2.41 of A_260/230_) (Figure 2c and Table 3).

### 3.2 Time and cost effectiveness of methods

The most cost-effective method was DBT (6.10 EUR per sample), while the most expensive method, MCB (13.50 EUR per sample), cost 121% more per sample. The MMV and MMM methods were 32.6 and 34.8% more expensive than DBT, respectively. The hands-on time required to perform 12 reactions was not influenced by the Gram classification of the strains in any of the tested methods. The DBT and MMV methods required the least hands-on time (83 min and 86 min, respectively), whereas the MMM kit took more than twice as long (169 min). The MCB kit took 51 min longer than the fastest protocol. Overall, the DBT kit was the quickest method, both including and excluding the incubation step for Gram-negative and Gram-positive samples (Table 3).

### 3.3 Sequencing statistics

**Figure 3:**
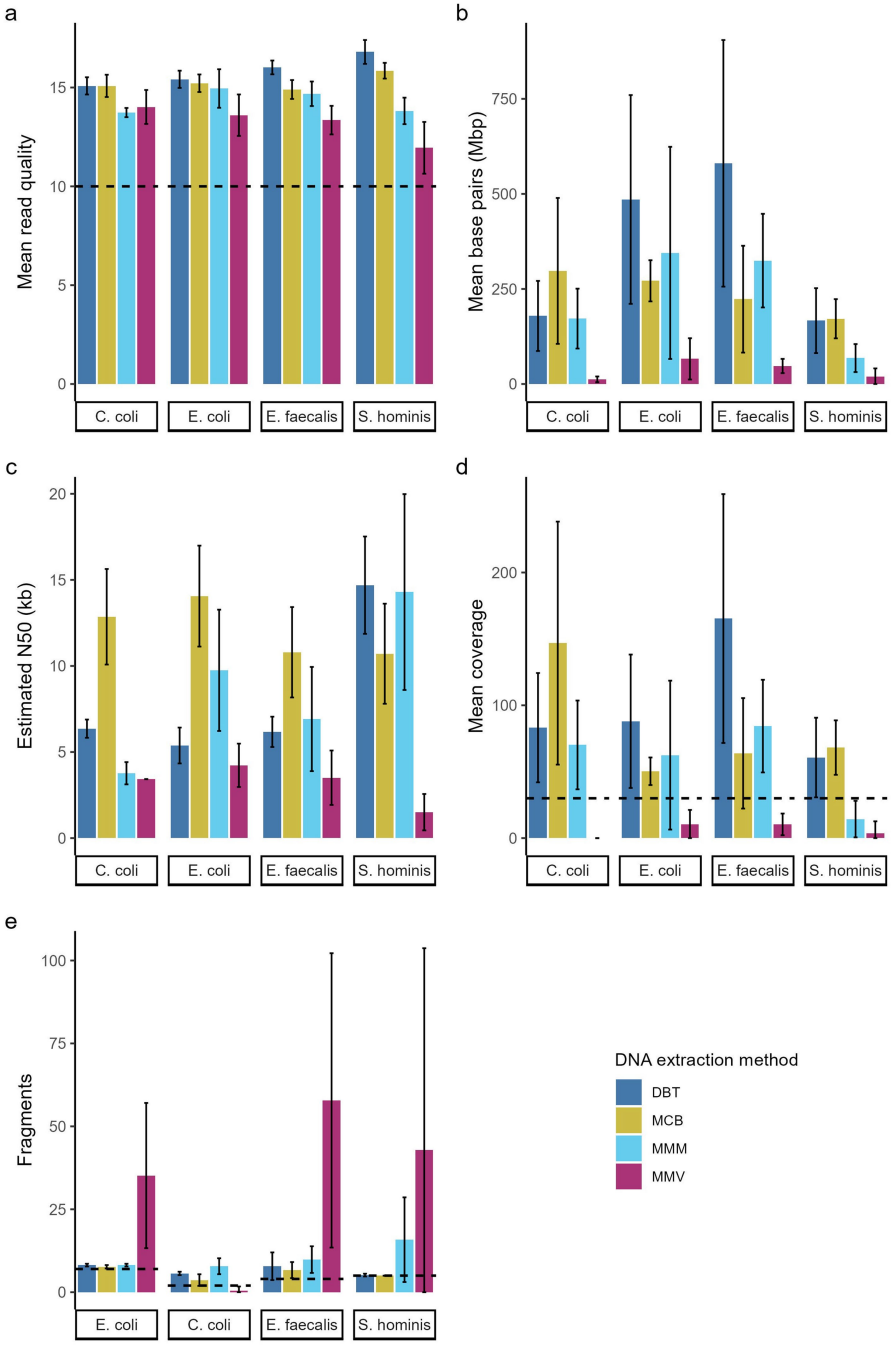
Effect of DNA extraction methods. **(a)** Mean quad quality (Q-score), **(b)** number of base pairs (bp), **(c)** estimated N50 (kb), **(d)** mean sequencing depth, and **(e)** number of fragments in assembly of four bacterial species sequenced on the Nanopore sequencing platform. The bar charts show mean across replications ± SD of three independent replication that was sequenced in duplicates (*n* = 6). **(a)** Dotted line indicates the min. Read quality filtered for and **(d)** indicates minimum mean sequencing depth defined as adequate, and in this study. **(e)** Dotted line indicates the number of fragments (chromosome and plasmids) for each isolate according to closed reference genome. DNeasy Blood & Tissue spin-columns (DBT, dark blue), Monarch® HMW DNA Extraction kit for Tissue (MCB, yellow) MagMAX™ Microbiome Ultra Nucleic Acid Isolation kit (MMM, light blue), and MagMAX™ Viral/Pathogen Ultra Nucleic Acid Isolation kit (MMV, purple).

Pooled libraries of 24 samples were sequenced in duplicates on the GridION platform using R10.4.1 flow cells and run quality metrics were generated with Nanostat (Figure 1). The extraction method significantly influenced read quality for all strains (*p* < 0.02). The DBT and MCB methods yielded higher mean quality scores across the species (15.8 ± 0.8 and 15.3 ± 0.6, respectively), in comparison to the MMM and MMV methods. Notably, MMV yielded markedly lower quality scores for the Gram-positive strains *S. hominis* and *E. faecalis* (11.9 ± 1.3 and 13.4 ± 0.7, respectively) (Figure 3a and Table 3).

The MCB method showed the highest read N50 overall (12,105 bp), followed by MMM (N50 = 8,681) and DBT (N50 = 8,151 bp), all exceeding 8,000 bp. In contrast, MMV produced shorter read N50 values. The MMV method was significantly lower than MCB (*p* < 0.04) across all species and significantly lower than DBT and MMM for the Gram-positive strains (*p* < 0.05). The MMV method was significantly lower than DBT for *C. coli* (*p* = 0.007) and for the *E. coli* strain for the MMM method (*p* = 0.01) (Figure 3c and Table 3).

### 3.4 Evaluation of assembled genomes

Flye assemblies were evaluated based on sequencing depth and total genome length. Assemblies deviating by more than 10% from the corresponding closed reference genome were considered to have failed. Overall, MMV generated highly fragmented assemblies across all species. For MMV *C. coli* GENOMIC20-006 replicates only one sample assembled (1/6, 16.67%). The assembly showed a deviation of −98.88% relative to the reference, suggesting that only a small portion of the genome was recovered. The other methods, MCB, DBT, and MMM, produced relatively few fragments, except in the case of *S. hominis* SHOMINIS processed with MMM, where assemblies were more fragmented (16 ± 13) (Figure 3e). The mean depth varied substantially among methods, ranging from 6 ± 9× for MMV to 99 ± 68× for DBT. When filtered for a minimum mean depth of 30×, defined as minimal requirement for ensuring quality assemblies in this study, only DBT yielded successful assemblies for all replicates of the four strains (24/24, 100%). MCB achieved nearly complete success (22/24, 91.67%), while MMM showed a reduced success rate (17/24, 70.83%), with most failures occurring among the *S. hominis* strain replicates (5/6, 83.33%). In contrast, the MMV method produced no successful assemblies (0/24, 0%) and performed significantly worse than all other methods across both Gram-negative and Gram-positive species (Figure 3d and Table 3).

### 3.5 Sequencing depth evaluation and genomic characterization

The minimum sequencing depth threshold of 30 × was established for genome assemblies. However, full detection of all AMR genes was achieved in a subset of samples despite mean depth falling below this threshold. Specifically, eight of the 33 low-depth assemblies (24.24%) demonstrated complete AMR gene detection, encompassing all MCB samples (2/2, 100%) and the majority of MMM samples (5/7, 71.43%). By contrast, only one MMV sample with mean depth <30× achieved full AMR gene detection (1/24, 4.16%). Among assemblies that satisfied the quality threshold, only one single *E. coli* sample processed using the MMV method failed to detect all AMR genes (1/63, 1.59%). This sample produced an incomplete MLST profile. Upon increasing the minimum sequencing depth threshold to 50×, all AMR genes were successfully detected in every qualifying assembly (0/43, 0%).

### 3.6 Scoring system for evaluating WGS

A composite scoring system was applied to define successful WGS, requiring mean depth of ≥30×, complete MLST profile, and detection of AMR genes present in the reference genomes. Significant differences were observed among the DNA extraction methods (likelihood ratio test: χ^2^ = 70.3, df = 3, *p* < 0.0001), with the strain did not significantly influencing WGS success (χ^2^ = 4.9, df = 3, *p* = 0.18). DBT yielded complete WGS success across all replicates (24/24, 100%). MCB produced slightly lower success rate (21/24, 87.5%), although the differences were not statistically significant (OR = 0.13, 95% CI 0.001–1.40, *p* = 0.099). MMM showed significantly reduced performance (17/24, 70.83%, OR = 0.05, 95% CI 0.00036–0.44, *p* = 0.003) and performed particularly poorly for the *S. hominis* strain (1/6, 16.7%). The MMV method did not yield any successful WGS results (0/24, 0%, OR = 0.0004, 95% CI 0.0000013–0.0087, *p* < 0.001) and was significantly inferior to all other methods.

## 4 Discussion

In terms of startup costs, requirements for laboratory environment, and throughput, ONT’s MinION offers portable WGS uniquely suitable for AMR and outbreak surveillance in low-resource laboratories, especially as compared to other technologies such as Illumina and PacBio (Aruhomukama, 2022; Lu et al., 2022). However, DNA extraction remains a crucial step for high-quality sequencing and must yield high-quality DNA for relevant pathogens, be cost-effective, easy to perform, and feasible in resource-limited environments. This study evaluated the performance of four commercially available DNA extraction kits for ONT sequencing in the context of AMR surveillance in low resource settings (Table 2). Extractions were performed on four strains representing both Gram-positive and Gram-negative species aiming to identify a DNA extraction method applicable to most bacterial species (Table 1).

### 4.1 Challenges in cell lysis and DNA extraction across bacterial species

Our study demonstrated that the DBT and MCB methods produced enough DNA (≥200 ng per sample in a concentration of ≥20 ng/μl) across all four strains for library preparation using the SQK-RBK114 kit, following the most recent recommendations from ONT (RBK_9176_v114_revR_04Jun2025). The MCB method employed a high concentration of lysozyme (10 mg/ml) for Gram-positive lysis and showed the most effective performance for the *S. hominis* strain. In contrast, the MMM method failed to yield adequate DNA quantity for that strain, likely due to the species’ hard-to-lyse properties. *S. hominis* exhibits resistance to lysozyme and partial resistance to lysostaphin, contributing to its hard-to-lyse properties. These factors made the *S. hominis* strain relevant for inclusion in this study (Otto, 2013; de Oliveira et al., 2014; Pushkaran et al., 2015). Further, coagulase-negative staphylococci are increasingly recognized for their infectious potential and their potential role as a reservoir of methicillin-resistance, highlighting the importance of optimized DNA extraction for accurate identification (Otto, 2013; Xu et al., 2018).

Interestingly, MMM yielded the highest concentration for *E. coli* GENOMIC22-004 at 397.33 ± 92.65 ng/μl (Figure 2a), likely due to species-dependent susceptibility to bead-beating, which has been shown in prior studies (de Boer et al., 2010; Lim et al., 2018; Gryp et al., 2020; Zhang et al., 2021; Fernández-Pato et al., 2024; Kruasuwan et al., 2024). The MMV method, which employed a proprietary enzymatic mix containing lysozyme at an unknown concentration, did not yield sufficient DNA for library construction with the SQK-RBK114 kit with the newest ONT recommendations. These findings highlight that, particularly for hard-to-lyse Gram-positive species, additional enzymatic lysis (e.g., lysozyme ± lysostaphin for *Staphylococcus* spp.) and careful handling to minimize shear materially affect ONT suitability, even if these reagents are generally not supplied in the commercial kits.

### 4.2 DNA purity and yield: impacts on nanopore sequencing quality

ONT recommends using DNA samples with an A_260/280_ ratio of ~1.8 and an A_260/230_ ratio in the range of 2.0–2.2 for sequencing, although A_260/280_ and A_260/230_ ratios >1.8 are generally accepted for down-stream applications (Koetsier and Cantor, 2019). Our study found that the two magnetic bead-based methods, MMM and MMV, yielded DNA of lower purity compared to the other methods, suggesting possible protein contamination. However, in the *S. hominis* strain replicates, low DNA concentrations across several samples may also have contributed to the low A_260/280_ ratio, as concentrations below 20 ng/μl decreases the accuracy of measurements and should be interpreted with caution (Koetsier and Cantor, 2019). Methods MCB and DBT produced A_260/280_ ratios >1.8 in all replicates across the four species; however, multiple measurements for DBT and specifically the *C. coli* strain measurements for MMM exceeded 2.0, consistent with RNA carryover and/or DNA degradation signals (Demeke and Jenkins, 2010).

The low A_260/230_ ratios indicate contamination with proteins, lysis buffer, or phenols. The MCB and DBT methods exhibited lower ranges for the *E. coli* strain (Figure 2c) compared with the other strains, which suggests a strain-inherent factor affecting the extractions with both methods. While DBT showed significantly higher A_260/230_ ratios than the magnetic bead-based methods, the Gram-positive samples produced slightly lower ratios and showed reduced reproducibility. This could indicate incomplete lysis, as silica spin-columns are prone to clogging when overloaded or when samples are viscous, potentially leading to insufficient wash steps (Katevatis et al., 2017; Shetty, 2020). MCB had acceptable ranges for all Gram-positive and *C. coli* strain replicates (2.14–2.25 of A_260/230_), with minor indications of some RNA contamination or DNA degradation for some replicates (Figures 2b,c). The observed A_260/280_ and A_260/230_ ratios suggest a positive effect of RNase A treatment in reducing RNA contamination. This step was included only in the MCB method, which may explain its improved purity metrics.

### 4.3 Operational feasibility in low-resource settings

Accurate estimation of bacterial cell density is typically achieved by measuring OD600 from pure cultures using a spectrophotometer, which is more accurate than volume-based techniques such as inoculation loops (Jacobs et al., 2000; Beal et al., 2020). To reflect expected conditions in low-resource laboratories where spectrophotometers are often unavailable, we instead quantified the starting material using a sterile loop from streaked cultures on solid media. Magnetic beads-based purification methods may be more sensitive to inaccuracies in biomass quantification, potentially contributing to the variability in DNA yield and purity observed. The use of loop-based biomass estimation may therefore have contributed to the poor reproducibility observed in several quality metrics. Furthermore, the laboratory scientist was given only two practice runs per method prior to the study. This limited training possibly contributed to the high variability observed between many replicates and the general low reproducibility of some methods. Such variability due to user handling has been reported in the literature, particularly regarding methods with multiple technical steps (Gill et al., 2025; Taylor et al., 2025).

Overall, the magnetic bead-based methods MMM and MMV did not require non-standard equipment and had a relatively low cost per sample (~8 EUR); however, the MMM method was labor-intensive (Table 3).

This study found the investigated magnetic bead-based kits MMM and MMV unsuitable for AMR surveillance using WGS in settings where instruments like spectrophotometers may be unavailable and where laboratory staff may have limited time. The glass bead-based MCB method consistently produced high-quality DNA in sufficient quantities with good reproducibility. However, it was the most expensive (13.50 EUR per sample) and required access to a refrigerated centrifuge, which may not be available in low-resource microbiology laboratories. Furthermore, the MCB method involves isopropanol precipitation, posing chemical hazards and requiring use of a laminar flow bench during parts of the workflow (Table 2). Considering these factors, we propose the DBT method as the most suitable for AMR surveillance in low-resource settings. Notably, it required the least hands-on time (83 min for 12 reactions) and was the most cost-effective method at 6.10 EUR per sample (Table 3). Furthermore, the DBT kit has been widely used across Africa and South and Southeast Asia for DNA extraction during various outbreaks including methicillin-resistant *Staphylococcus aureus* (MRSA)(Dayie et al., 2019; Obanda et al., 2022; Khoothiam et al., 2023; Ngoubangoye et al., 2023; Islam et al., 2024). The accessibility and versatility of the DBT kit are particularly important in regions such as Africa, which face frequent supply chain challenges for laboratory consumables (Petti et al., 2006; Ejekam et al., 2023; Mukhwana et al., 2024).

### 4.4 Whole genome sequencing success

All four DNA extraction methods assessed in this study incorporated different combinations of cell lysis and DNA purification techniques: beads-beating, chemical, and enzymatic lysis methods were used in conjunction with magnetic bead, silica spin-column, or glass bead-based purification strategies (Table 2). When comparing MMV and MMM, both using magnetic bead purification, bead-beating in MMM did not appear to excessively shear DNA, contrary to observations in other studies (Figures 2b,c, 3b and Table 3) (de Boer et al., 2010; Kruasuwan et al., 2024; Purushothaman et al., 2024).

The MCB method was the only HMW method included, designed specifically for long-read sequencing. It demonstrated improved DNA purity and produced longer read N50 compared to the other methods. However, it did not result in consistently higher mean sequencing depth. For Gram-negative species, both DBT and MCB methods generated successful assemblies for all replicates, while MMM exhibited poor reproducibility for the *E. coli* strain. MMV yielded significantly less data per sample and shorter read N50, resulting in no successful assemblies (Figures 2b,c, 3b–d and Table 3).

### 4.5 Sequencing depth threshold for reliable AMR surveillance

In this study, a sequencing depth threshold of 30× was applied. Nevertheless, full detection of all AMR genes was observed in 24.24% of the assemblies with depths below this threshold. By contrast, assemblies with mean depth ≥30× exhibited complete detection of AMR genes and MLST loci in all but one sample. This finding is further supported by the observation that increasing the minimum sequencing depth threshold to 50× led to complete detection of AMR genes and MLST loci in all assemblies that passed quality control. These results suggest that a 30× threshold may be insufficient for consistently producing high-quality assemblies and that a higher threshold could enhance reliability. However, increasing the threshold to 50× would exclude a substantial proportion of assemblies (20/62, 30.65%) in which both AMR genes and MLST loci were successfully detected, potentially leading to unnecessary re-sequencing of otherwise high-quality assemblies and reducing overall cost effectiveness. Consequently, MLST typing, when used in conjunction with other quality control metrics, may serve as a robust framework for evaluating assembly quality. This is particularly relevant in a surveillance context, where reference genomes are not available and the completeness of AMR gene detection cannot be directly verified. In contrast, MLST typing offers a more interpretable signal of assembly quality, as failure to assign a known MLST type may indicate insufficient coverage or assembly errors. As such, incorporating MLST into the QC process may allow the retention of informative assemblies without necessitating an increase in the sequencing depth threshold. These findings align with previous studies indicating that a mean depth of 30×, combined with long read lengths, is generally sufficient for *de novo* genome assembly and single-nucleotide variant (SNV) detection in species such as *E. coli*, *Staphylococcus aureus*, and *Enterococcus faecium* (Kruasuwan et al., 2024; Purushothaman et al., 2024). Nonetheless, other studies report that 50× may be necessary, depending on read N50 and genome size (De La Cerda et al., 2023; Wu et al., 2023).

### 4.6 Recommendations for routine AMR surveillance in low-resource laboratories

To define successful WGS, we developed a scoring system based on three criteria: sequencing depth ≥30×, detection of all AMR genes, and detection of all MLST loci. Although the MCB method produced the longest read N50, the DBT method achieved higher mean sequencing depth and 0% composite failures, whereas MCB showed a failure rate of 12.5%. The high WGS success rate observed for DBT aligns with findings from Eagle et al., who evaluated five commercial kits for ONT sequencing of *Salmonella*. In their study, DBT demonstrated the best performance in terms of cost-efficiency, DNA yield, and overall sequencing performance (Eagle et al., 2023). The magnetic bead-based methods, MMM and MMV performed significantly worse. MMV achieved a 0% success rate, while MMM showed a particular poor performance for especially the *S. hominis* strain.

While there was no significant difference in WGS performance between DBT and MCB, the DBT method had a clear advantage in cost-efficiency and practicality, being less than half the cost of MCB and requiring less auxiliary equipment. Altogether, these findings support the use of the DBT method as a robust and scalable DNA extraction approach for ONT-based WGS in low-resource settings, particularly for pathogen and AMR surveillance across a diverse range of bacterial species.

### 4.7 Limitations and potential bias

A key limitation of this study is that all DNA extractions were performed by a single laboratory scientist, who may have had varying proficiency across the different methods. This introduces potential operator bias, as performance could partially reflect individual ease-of-use rather than the intrinsic quality of the kits themselves. To mitigate this effect, the laboratory scientist received standardized training in all four methods. An additional source of bias arose from the library preparation design, in which extractions from two methods were pooled on the same flow cell: DBT was paired with MMV, and MCB with MMM. Although sequencing was performed in duplicate, this pairing may have affected downstream performance. It is possible that suboptimal DNA from one method could have negatively impacted the performance of the co-sequenced method during the multiplexed ONT sequencing.

Only four different bacterial strains were included in the study, which may not represent the full diversity of Gram-positive and Gram-negative species relevant to AMR surveillance. In addition, each extraction was performed in triplicate, which may not fully capture the variability in low-resource settings.

## Data Availability

The datasets presented in this study can be found in online repositories. Raw sequencing reads have been deposited in the Sequence Read Archive (NCBI) under BioProject accession number PRJNA1328464. The accession numbers for individual samples are available in the article/Supplementary Material.
