# Supplementary material for: Evaluation of four DNA extraction kits for implementation of nanopore sequencing in routine surveillance of antimicrobial resistance in low-resource settings
Source: Front Microbiol. 2025 Nov 25;16:1715467. doi: 10.3389/fmicb.2025.1715467 (PMC12685845; doi:10.3389/fmicb.2025.1715467)
Supplement: Supplementary file 2 [file Table_2.pdf]

Table S2: Nanopore sequence run metrics

Flowcell number	Methods on run	Library DNA concentration	Active pores	Total data produced (GB)
FAW16519	DBT - MMV	43.2	1253	53.71
FAW16577	DBT - MMV	37.6	1299	121.11
FAW16614	MCB - MMM	63.6	1480	110.15
FAW16625	MCB - MMM	58.4	1390	148.12

Bases called Pass (Gb)	Bases called Fail (Gb)
3.07	0.69425
7.14	1.36
4.69	1.87
7.53	2.45
